# The Effect of Hydroalcoholic Extract of *Salvia miltiorrhiza* on Hormonal and Cellular Parameters of Spermatogenesis in Male Rats After the Consumption of Ibuprofen

**DOI:** 10.1002/fsn3.70705

**Published:** 2025-07-29

**Authors:** Fatemeh Salehi, Leila Zarei, Yaser Mokhayeri, Omid Rajabzadeh

**Affiliations:** ^1^ Department of Anatomy Sciences, Faculty of Medicine Lorestan University of Medical Sciences Khorramabad Iran; ^2^ Center for preventive Neurology, Wolfson Institute of population health Queen Mary university of London London UK

**Keywords:** FSH, ibuprofen, LH, oxidative stress, *salvia miltiorrhiza*, sperm parameters, testosterone

## Abstract

*Salvia miltiorrhiza* (SM), a medicinal herb known for its antioxidant and anti‐inflammatory properties, has been traditionally used to treat various physiological disorders. This study aimed to evaluate the potential protective effects of SM extract on spermatogenesis in male Wistar rats following ibuprofen administration. A total of 42 adult male Wistar rats were randomly divided into six groups: control group; SM group (200 mg/kg); ibuprofen‐treated groups at doses of 15 mg/kg (IbL) and 30 mg/kg (IbH); and two co‐treatment groups receiving SM (200 mg/kg) along with either 15 mg/kg or 30 mg/kg of ibuprofen. Serum levels of testosterone, FSH, and LH were measured using ELISA assay kits. Oxidative stress markers including TAC, MDA, GPx, and CAT were also assessed using specific commercial diagnostic kits according to the manufacturer's instructions. Histopathological evaluation of testicular tissue was performed using the Johnsen scoring system, and sperm parameters were analyzed from the epididymal tail. Significant histopathological changes were observed only in the IbH group (30 mg/kg), showing a decrease in seminiferous tubule thickness and count compared to control (*p < 0.05*). No significant differences were found in Johnsen scores or necrosis grades among groups. Serum testosterone levels significantly increased in the SM‐treated group (*p <* 0.05), while FSH and LH levels remained unchanged. The highest CAT activity was observed in the SM group, whereas the lowest was in the IbH and IbL groups (*p <* 0.05). The highest MDA level was detected in the IbL group, and the highest GPx level was seen in the SM + IbH group. Sperm parameters improved significantly in the SM‐treated groups but were adversely affected by high‐dose ibuprofen (*p* < 0.05). These findings indicate that ibuprofen, especially at a high dose, may impair spermatogenesis and sperm quality, potentially through induction of oxidative stress. In contrast, SM demonstrated protective effects by improving testosterone levels, enhancing antioxidant defense (TAC), and partially restoring sperm parameters. These results suggest that SM could serve as a potential natural supplement to mitigate the adverse effects of ibuprofen on male reproductive health.

## Graphical abstract

SM extract could be able to counter inflammation owing to the extra consumption of popular drugs such as ibuprofen.

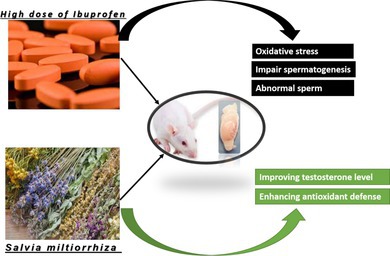

**Abbreviations**
CAT: catalase
FSH: follicular stimulating hormone
GECE: highest number of germinal cell layers
GECT: germinal epithelium cell thickness
GPx: glutathione peroxidase
IbH: ibuprofen high dose
IbL: ibuprofen low dose
LH: luteinizing hormone
MDA: malondialdehyde
MSTD: mean seminiferous tubule diameter
SM: Saliva miltiorrhiza
TAC: total antioxidant capacity

## 1 Introduction

Infertility, defined as the inability to achieve pregnancy after 1 year of unprotected intercourse, affects approximately 10%–15% of couples globally, with male factors contributing to nearly half of all cases (Esteves 2014; Agarwal et al. 2019). Male infertility is often associated with impaired spermatogenesis, reduced sperm quality, and increased oxidative stress, which can lead to DNA damage in sperm cells (Semet et al. 2017). Nonsteroidal anti‐inflammatory drugs (NSAIDs), including ibuprofen, are among the most commonly used medications worldwide for their analgesic, antipyretic, and anti‐inflammatory effects (Wongrakpanich et al. 2018). However, recent studies have raised concerns about their potential adverse effects on male reproductive function. Ibuprofen has been shown to disrupt testicular hormone production, particularly testosterone, and negatively affect sperm parameters in both human and animal models (Kristensen et al. 2018; Rossitto et al. 2019). These effects may be mediated through multiple mechanisms, including suppression of prostaglandin and testosterone synthesis, inhibition of nitric oxide production, and zinc ion chelation (Banihani 2019). *Salvia miltiorrhiza* (SM), a medicinal herb belonging to the Lamiaceae family, contains various bioactive compounds such as flavonoids, diterpenes, saponins, and vitamins C and E (Lu and Foo 2002). It has long been used in traditional medicine for its cardiovascular, hepatic, and renal protective properties. Recent studies have confirmed its antioxidant, anti‐inflammatory, and antimicrobial activities (Sulniute et al. 2016; Abu‐Darwish et al. 2013). Importantly, SM has been reported to enhance Leydig cell activity and testosterone secretion by strengthening the body's antioxidant defense system (Jambor et al. 2020). Assessment of serum levels of key reproductive hormones—including testosterone, luteinizing hormone (LH), and follicle‐stimulating hormone (FSH)—is essential for evaluating testicular function and identifying potential disruptions in spermatogenesis. These hormones not only regulate sperm production but also serve as important biomarkers for diagnosing endocrine disorders and guiding therapeutic interventions (Fontana et al. 2024). In this study, we assessed these hormonal parameters alongside oxidative stress markers such as total antioxidant capacity (TAC), malondialdehyde (MDA), glutathione peroxidase (GPx), and catalase (CAT). These enzymes and metabolites play critical roles in maintaining redox homeostasis within testicular tissue, and their imbalance has been strongly linked to impaired spermatogenesis and poor semen quality. Despite the widespread use of ibuprofen and its potential impact on male fertility, limited research has focused on identifying natural agents that could mitigate these effects. While several plant extracts have demonstrated protective effects against drug‐induced reproductive toxicity (Akhigbe et al. 2024; Walker 2024), few studies have specifically investigated the role of natural plants like *Salvia miltiorrhiza* (Davoodi et al. 2020) in preserving male reproductive function following NSAID exposure. To our knowledge, this is among the first studies to comprehensively evaluate the protective effects of *Salvia miltiorrhiza* extract against ibuprofen‐induced alterations in hormonal profiles, oxidative stress markers, testicular histopathology, and sperm parameters in male Wistar rats. Our findings may provide novel insights into the potential use of SM as a complementary agent in preventing or reducing the adverse effects of NSAIDs on male reproductive health.

## 2 Materials and Methods

The impact of animal testing was conducted by the Animal Ethics Committee at the Ethics Committee of Lorestan University of Medical Sciences (IR.Lums.REC.1401.142). The present research is of the “experimental‐laboratory” type, during which the treated samples were compared to the control group.

Sample preparation: 42 adult male Wistar rats with an average weight of 200–300 g (10–12 weeks old) were obtained from the laboratory animal breeding center located at the Razi Medical Sciences Research Center in Lorestan. Animals were kept in a special room at a temperature of 25°C (humidity about 65%) with photoperiod of 12 h. Water and pellet diet were provided to the rats without limits. Before starting the experiment, animals were kept in laboratory conditions (1 week), were randomly grouped, and the samples in each group were numbered. None of the animals showed any signs of illness or evidence of disease in appearance during the grouping.

Grouping: The rats were randomly divided into six groups (*n* = 7) as follows:

Control (CO): recipient normal food and water.

SM: recipient of sage extract at a dose of 200 mg/kg.

IbL: recipient of ibuprofen at a dose of 15 mg/kg.

IbH: recipient of ibuprofen at a dose of 30 mg/kg.

SM + IbL: recipient of sage at a dose of 200 mg/kg and ibuprofen 15 mg/kg.

SM + IbH: recipient of sage at a dose of 200 mg/kg and ibuprofen 30 mg/kg (Ahmadi et al. 2013; McQuay and Moore 2007; Davoodi et al. 2020).

### 2.1 Preparation and Injection of the SM Extract

Fresh leaves of *Salvia miltiorrhiza* were collected from the research farm of Barij Essence Company, located in Kashan, Iran. A voucher specimen was deposited at the Department of Agriculture, Barij Essence Research Center, under herbarium number 186‐1 for taxonomic identification and future reference. For extract preparation, 500 g of fresh leaves were shade‐dried at room temperature (25°C) for 10 days. The dried plant material was then ground into a fine homogeneous powder using a laboratory mill. The powdered sample was subsequently macerated in 1 L of 96% ethanol in a sealed glass container and left at ambient temperature for 48 h with daily manual shaking (15 min) to ensure complete extraction. Following maceration, the mixture was filtered through filter paper to remove particulate matter. The solvent was then removed under reduced pressure using a rotary evaporator (IKA RV 05 Rotary Evaporator, Iran). This process yielded approximately 100 g of viscous semi‐solid extract, which was further concentrated by drying in a vacuum oven for 24 h. Finally, the resulting dried extract was reconstituted in distilled water to achieve the desired concentration and sterilized by filtration (0.22 μm membrane filter) prior to experimental use. Prior to solvent removal, physicochemical analysis of the *Salvia miltiorrhiza* liquid extract was carried out in the central laboratory of Barij Essence Pharmaceutical Co., Iran (Reference Code: FCL64‐03). The analytical results are summarized as follows:

Specific Gravity: 0.974 g/mL (Method: USP38 <h841i>).

pH Value: 5.83 (Method: ISIRI 1487, Part 2, Section 2.4).

Dry Residue: 12.83% w/w (Method: BP2015).

Refractive Index: 1.3764 (nD) (Method: ISIRI 2274‐6).

Rosmarinic Acid Content: 14.28 mg/mL (Barij Essence Internal Reference Method). The total antioxidant capacity of the hydroalcoholic extract of *Salvia miltiorrhiza* was evaluated using the Ferric Reducing AntioxidantPower (FRAP) assay (Ahmadi et al. 2013; Davoodi et al. 2020).

Animal care method: For 21 days, ibuprofen was administered to the rats, and for 60 days, hydroalcoholic extract of SM was gavaged to them. The doses of gavage were administered once a day at 10 AM.

Preparation of Ibuprofen: Ibuprofen tablets from Alborz Darou Company were prepared, and based on the weight of the rats, doses of 30 mg/kg and 15 mg/kg were formulated. DMSO 5% ibuprofen was selected.

Surgical procedure: The rats were anesthetized (10% ketamine and 2% xylazine injection via the intraperitoneal method xylazine 10 mg/kg + ketamine 80 mg/kg) and placed in a supine position on the surgical table. Under sterile conditions, blood samples were collected from the left ventricle of the heart using a 5/0 syringe. Immediately following blood collection and transfer to serum separator tubes, the scrotal area was shaved with clippers. Subsequently, a midline incision was made through the skin and peritoneum of the scrotal region using a scalpel. The testis and epididymis were carefully excised, rinsed with saline, and weighed individually. The testis was bisected along the longitudinal axis, and each half was placed separately into designated containers for histological and biochemical analyses. The caudal portion of the epididymis was transferred to a pre‐warmed container containing RPMI‐1640 culture medium for assessment of sperm parameters.

Hormonal assessment: The collected blood was centrifuged for 10 min at 8000 rpm and the separated serum were kept at −20°C. ELISA assay was performed for evaluation of FSH, LH (FSH; Pishtaz Teb, Iran; catalog number: PT‐FSH‐96), LH (Pishtaz Teb, Iran; catalog number: PT‐LH‐96). The assays performed with standard protocol according to the manufacturer's instructions and an ELISA reader apparatus. (Stat fax 3200, USA).

Histological studies: For histopathological analysis, the left testis was sectioned longitudinally and subsequently fixed in a 10% neutral‐buffered formalin solution (prepared by mixing 18 mL of distilled water with 2 mL of 37%–40% formaldehyde) for tissue preservation, staining, and microscopic examination. Following standard tissue processing protocols, including dehydration, clearing, and paraffin embedding, 5‐μm‐thick sections were obtained using a rotary microtome. Hematoxylin and Eosin (H&E) staining was performed to assess testicular histomorphometry under a light microscope. In parallel, the contralateral testis was immediately preserved at −80°C for subsequent biochemical analyses, particularly to evaluate oxidative stress markers and enzymatic activity.

Histological assessment of spermatogenesis was performed using the Johnson scoring system, a semiquantitative method that grades spermatogenesis on a scale from 1 to 10 based on the presence and maturation of germ cells within seminiferous tubules. Higher scores reflect more advanced and complete spermatogenesis.

A pathologist who was unaware of the groupings and tests evaluated all the microscopic slides. A light microscope with magnifications of 40, 100, and 200 was used to estimate histopathological damage.

To evaluate key histological parameters, including the mean seminiferous tubule diameter (MSTD) and the mean germinal epithelium cell thickness (GECT) in micrometers, a calibrated ocular micrometer was employed under light microscopy. For each histological slide, measurements were obtained from four distinct regions of the tissue section. In each region, 10 seminiferous tubules were randomly selected and analyzed to ensure representative sampling.

### 2.2 Pathological Examination

Histological changes and testicular necrosis were assessed using a histological grading method classified into 4° as follows (Cosentino et al. 1986):

Grade I: Natural structure of the tissue and organized arrangement of germinal cells without tissue necrosis and bleeding.

Grade II: Testicular damage characterized by reduced cellular organization and diminished intercellular adhesion within the germinal epithelium, along with mild hemorrhage and focal necrosis.

Grade III: Testicular tissue alterations including cellular degeneration, irregular arrangement of germinal cells, and nuclear pyknosis, along with a diminished distinction between the borders of seminiferous tubules and the presence of extensive interstitial hemorrhage.

Grade IV: The testicular tissue exhibited severe damage, characterized by complete occlusion of seminiferous tubules, coagulative necrosis of germinal cells, and extensive hemorrhage.

### 2.3 Sperm Analysis

To evaluate sperm parameters, the cauda epididymis was excised and finely minced in a Petri dish containing 5 mL of RPMI 1640 culture medium. The tissue suspension was then incubated at 37°C for 15 min to allow sperm release into the medium. Sperm parameters including concentration, motility, viability, and morphology were analyzed according to the guidelines provided by the World Health Organization (WHO 2021).

### 2.4 Sperm Motility Assessment

To evaluate sperm motility, a 5 mL aliquot of the epididymal suspension was diluted in RPMI 1640 culture medium. A small drop of the diluted sample was placed on a pre‐warmed glass slide and immediately examined under a phase‐contrast microscope at 400× magnification. At least 200 sperm cells were assessed per sample to minimize measurement error and ensure reliable quantification of motility parameters.

Preparation of a 1%–90% solution: Dissolve 9 g of NaCl in 100 mL of distilled water.

2%–5%, Y Eosin: (5/0 g of eosin in 100 mL of 9% sodium chloride).

Five microliters of diluted sperm sample was placed on a glass slide along with 5 μL of reagent solution, allowed to sit for 30 s, and then evaluated under a microscope.

Sperm viability assessment (eosin staining method): The second parameter that should be quickly assessed after motility is sperm viability. This parameter was evaluated using the eosin staining technique, as described in the World Health Organization (WHO) Laboratory Manual for the Examination and Processing of Human Semen (5th edition, 2010). This method operates on the principle that viable spermatozoa, possessing intact plasma membranes, are capable of repelling the dye, while non‐viable spermatozoa, with damaged membranes, absorb eosin and become visibly stained under light microscopy.

Sperm counting: To determine sperm concentration, a fixative was first prepared to kill and stabilize the sperm, so that their motility would be eliminated and counting could be facilitated.

Sperm morphology assessment: For the evaluation of sperm morphology, a 10 μL aliquot of the diluted sperm suspension was placed onto a clean glass slide, and a smear was created by using a second slide in a standard spreading technique. The prepared smear was allowed to air‐dry completely and subsequently fixed in 70% ethanol. Following fixation, H&E staining was carried out to facilitate morphological analysis under light microscopy.

Evaluation of antioxidant enzyme activity: The testicular tissue samples stored at −80°C were thawed at room temperature, cut into small pieces, and homogenized in phosphate‐buffered saline (PBS) using a homogenizer. Following centrifugation, the supernatant was collected for biochemical analysis. The total protein content in the supernatant was determined using the Biuret method, which is based on the formation of a violet‐colored complex between copper ions and peptide bonds in an alkaline medium. Protein concentrations were calculated using a standard curve prepared with bovine serum albumin (BSA), and all results were normalized to total protein content to ensure accurate comparison across samples.

Antioxidant enzyme activities, including malondialdehyde (MDA), total antioxidant capacity (TAC), catalase (CAT), and glutathione peroxidase (GPX), were assessed using commercial assay kits:

MDA (#ZB‐MDA‐96A, ZellBio GmbH Corporation, Germany).

TAC (#ZB‐TAC‐96A, ZellBio GmbH Corporation, Germany).

CAT (#ZB‐CAT‐96A, ZellBio GmbH Corporation, Germany).

GPX (#RS504, Randox Laboratories Ltd., Crumlin, UK).

All measurements were performed according to the manufacturers' instructions. To ensure accuracy and reproducibility, all assays were conducted in duplicate.

Statistical analysis: To examine and analyze the raw data obtained from this study, Stata software version 17 was used. The one‐way analysis of variance (ANOVA) method and the Scheffé post hoc test were used. The results are presented as the standard error mean, and a *p* value < 0.05 was considered significant.

## 3 Results

### 3.1 Testicular and Epididymal Weight

The comparative analysis of testis and epididymis weight among the groups in Table 1 indicates that different doses of ibuprofen and a single dose of SM have had a significant effect between some groups (*p* < 0.05). The highest testicular weight is related to the SM and has a significant difference compared to the CO. The lowest weight of the epididymis was observed in IbH, and no significant differences were found between any of the groups (*p* < 0.05).

**TABLE 1:** Comparison of testicular and epididymal weight. All values are displayed as Mean ± SD.

Groups	Testicular weight	Epidydimal weight
Co	1.25 ± 0.035	0.51 ± 0.018
SM	**1.39 ± 0.019** ^a^	0.51 ± 0.008
IbH	1.24 ± 0.048	0.49 ± 0.009
IbL	1.27 ± 0.073	0.5 ± 0.008
SM + IbH	1.22 ± 0.019	0.5 ± 0.007
SM + IbL	1.25 ± 0.063	0.5 ± 0.004

*Note:* Data are expressed as mean ± standard deviation (Mean ± SD). The testicular weight in the SM group was significantly higher compared to the control (Co) group (*p* < 0.05; indicated by superscript “a”). No notable differences were observed in either testicular or epididymal weights among the other treatment groups when compared to the control group.

^a^ *p* < 0.05.

### 3.2 Johnson Scoring Results

The Johnson score findings indicated that there were no statistically significant differences in spermatogenesis stages between the ibuprofen and SM groups and the control group. Therefore, ibuprofen did not exhibit a negative impact on the progression of germ cell development during spermatogenesis (*p* < 0.05) (Table 2).

**TABLE 2:** Comparison of pathological parameters using Johnson's method.

Groups	VGO	GO	NG
Co	0 ± 0.00	0 ± 0.00	0 ± 0.00
SM	0.642 ± 0.852	0 ± 0.00	0 ± 0.00
IbH	0.5 ± 0.645	0.785 ± 1.21	0 ± 0.00
IbL	0.5 ± 0.866	0.585 ± 0.79	0 ± 0.00
SM ± IbH	0.64 ± 0.62	0.428 ± 0.73	0 ± 0.00
SM ± IbL	0 ± 0.00	0.685 ± 0.89	0.21 ± 0.56

*Note:* Data are expressed as mean ± standard deviation (Mean ± SD). The semiquantitative histopathological scores were categorized into three groups: Not Good Organization (NG, score ≤ 8), Good Organization (GO, score 9), and Very Good Organization (VGO, score 10), based on the cellular maturation within seminiferous tubules. *p* value < 0.05 was considered statistically significant.

### 3.3 Sperm Morphology

**FIGURE 1:**
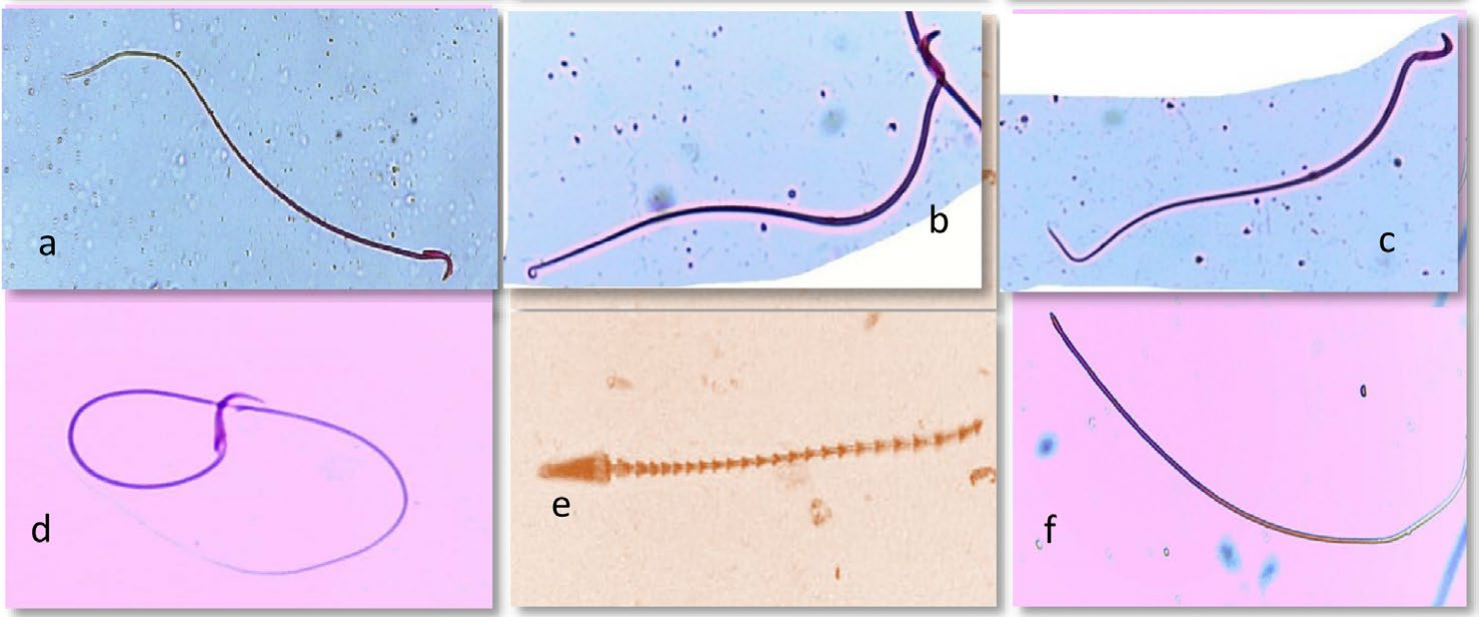
Different states of sperm morphology: (a) Normal sperm; (b) sperm with a twisted body; (c) bent tail; (d) short tail; (e) abnormal head and fragmented body; (f) non‐head sperm.

A comparative analysis of sperm morphology among the treatment groups receiving SM and ibuprofen versus the control group revealed statistically significant differences (*p* < 0.05). The highest number of abnormal sperm was observed in the groups treated with high‐dose ibuprofen (IbH), indicating a negative impact of this dose on sperm development and maturation. In contrast, the SM‐treated groups showed the highest percentage of normal sperm morphology, suggesting a protective effect of SM against structural abnormalities. The most common sperm abnormality was related to their tail. (Table 3 and Figure 1).

**TABLE 3:** Morphology of cauda epididymal spermatozoa. Data with normal distribution are displayed as Mean ± SD.

Group	*N* (%)	ST (%)	FT (%)	STT (%)
**CO**	84.72 ± 0.74	11.42 ± 1.09	3.88 ± 1.03	1
**SM**	**83.4 ± 1.22** ^a^	12.11 ± 1.28	4.47 ± 0.73	0.071 ± 0.18
**IbH**	**74.81 ± 0.90** ^a^	**17.54 ± 2** ^a^	**9.5 ± 2.08** ^a^	0 ± 0.00
**IbL**	81.32 ± 0.96	14.37 ± 1.49	2.74 ± 2.32	0 ± 0.00
SM + IbH	78.44 ± 2.85	11.67 ± 1.54	8.21 ± 3.55	0.0014 ± 0.0037
SM + IbL	**81.25 ± 3.10**	**13.29 ± 1.63**	**2 ± 1.59**	**0 ± 0.00**

*Note:* Data are expressed as mean ± standard deviation (Mean ± SD). Morphological abnormalities were categorized into three types: Stretching Tail (ST), Foil Sperm (FT), and Short Sperm Tail (STT), with the remaining percentage representing morphologically normal sperm (N). ^a^ *p* value < 0.05 was considered statistically significant. These findings suggest that high‐dose ibuprofen may impair sperm maturation and induce morphological abnormalities, particularly affecting tail structure.

^a^ *p* value < 0.05.

### 3.4 Sperm Motility

The findings of this study indicate that sperm motility was significantly improved in the SM group, which achieved the highest percentage of progressively motile sperm. In contrast, the groups treated with ibuprofen showed an increase in the percentage of immotile and static sperm (*p* < 0.05) (see Table 4). The highest percentage of immotile sperm was observed in the IbH and SM + IbH groups, which were statistically significant (*p* < 0.05).

**TABLE 4:** Sperm motility comparison. Data with normal distribution are displayed as mean ± SD.

Groups	MM (%)	M (%)	NM (%)
CO	7.6 ± 0.74	63.97 ± 1.5	28.42 ± 1.01
SM	6.8 ± 0.79	66.01 ± 1.25	26.88 ± 1.51
IbH	**19.48 ± 0.96** ^a^, ^b^	**56.07 ± 1.96** ^a^, ^b^	**24.5 ± 2.21** ^a^
IbL	**12.65 ± 0.94** ^a^, ^b^	**57.91 ± 2.31** ^a^, ^b^	29.6 ± 2.73
SM + IbH	**13.05 ± 1.17** ^a^, ^b^	64.22 ± 2.29	**22.78 ± 3.10** ^a^
SM + IbL	**9.07 ± 1.34** ^a^, ^b^	61.44 ± 2.23	26.64 ± 4.61

*Note:* Data are expressed as mean ± standard deviation (Mean ± SD). Sperm motility was classified into three categories: progressive motility (M), non‐progressive motility (MM), and immotile sperm (NM). These results indicate that high‐dose ibuprofen may negatively affect sperm motility by increasing non‐progressive movement and reducing progressive motility.

^a^ *p* < 0.05 compared with the control group.

^b^ *p* < 0.05 compared with SM group.

### 3.5 Sperm Viability and Concentration Results

*Salvia miltiorrhiza* (SM) demonstrated a significant positive effect on sperm viability and survival rates. Ibuprofen, at the high dose (IbH), resulted in the lowest percentage of viable sperm, and this difference was statistically significant (*p* < 0.05) (see Table 5). Based on these findings, it can be inferred that SM enhances sperm concentration (count), while the IbH and IbL groups exhibited the lowest sperm concentration. A statistically significant difference was observed between these two groups compared to CO and SM groups (*p* < 0.05).

**TABLE 5:** Comparison of sperm viability and concentration.

Groups	Viability (%)	Concentration (%)
CO	65.47 ± 0.56	43.68 ± 2.23
SM	66.01 ± 0.61	**45.50 ± 1.49** ^a^
IbH	**62.48 ± 1.33** ^a^	**41.63 ± 1.56** ^a^, ^b^
IbL	64.88 ± 0.68	**41.41 ± 0.71** ^a^, ^b^
SM + IbH	64.92 ± 0.53	43.59 ± 1.42
SM + IbL	65.18 ± 0.52	43.47 ± 1.25

*Note:* These findings suggest that high‐dose ibuprofen may impair both sperm viability and concentration, whereas low‐dose treatment or combination with SM intervention appears to mitigate these effects to some extent.

^a^ *p* < 0.05 compared with the control group.

^b^ *p* < 0.05 compared with the SM group.

### 3.6 Casentino Method

In the analysis of baseline damage scores obtained using the Casentino method, no statistically significant difference was observed between the CO group and the other experimental groups (*p < 0.05*). The highest percentage of healthy and non‐necrotic cells was detected in the CO group. These findings indicate that neither the SM treatment nor the different doses of ibuprofen exerted a significant effect on the Casentino histopathological grading or tissue damage (Table 6).

**TABLE 6:** Grading by the Casentino method.

Groups	MG	G	B	VB
CO	0 ± 0.00	0.92 ± 1.30	0 ± 0.00	0 ± 0.00
SM	0.5 ± 0.64	0.14 ± 0.37	0.64 ± 0.94	0.64 ± 1.31
IbH	0 ± 0.00	0 ± 0.00	1.71 ± 1.84	0 ± 0.00
IbL	0.42 ± 0.73	0 ± 0.00	0 ± 0.00	0 ± 0.00
SM + IbH	0 ± 0.00	0 ± 0.00	0 ± 0.00	0 ± 0.00
SM + IbL	0 ± 0.00	0 ± 0.00	0 ± 0.00	0.21 ± 0.56

*Note: p* < 0.05 compared with control group. MG: Grade II; G: Grade I; B: Grade III; VB: Grade IV.

### 3.7 Hormonal Outcomes

Regarding the serum levels of sex hormones—including luteinizing hormone (LH), follicle‐stimulating hormone (FSH), and testosterone—no statistically significant differences were observed in the levels of LH and FSH between the control group and the treatment groups receiving combinations of SM with either low‐ or high‐dose ibuprofen (SM + IbL and SM + IbH) (*p* < 0.05). However, a significant increase in serum testosterone levels was observed in the SM compared to other experimental groups (*p* < 0.05). This suggests that SM may have acted as an effective supportive factor in testosterone production, potentially by stimulating Leydig cells (Table 7).

**TABLE 7:** Comparison of sex hormones.

Groups	LH (mlu/mL)	FSH (mlu/mL)	Tes (mg/mL)
CO	0.11 ± 0.008	7.82 ± 0.33	1.11 ± 0.21
SM	0.107 ± 0.011	7.97 ± 0.55	**5.1 ± 0.16** ^a^
IbH	0.107 ± 0.0048	7.48 ± 0.46	1.1 ± 0.11
IbL	0.104 ± 0.07	7.31 ± 0.50	1.10 ± 0.19
SM + IbH	0.107 ± 0.004	7.58 ± 0.56	1.25 ± 0.25
SM + IbL	0.107 ± 0.007	7.55 ± 0.56	1.31 ± 0.32

*Note:* These findings suggest that while the SM was associated with elevated testosterone levels, neither ibuprofen administration nor its combination with SM had a major impact on the hypothalamic‐pituitary‐gonadal axis activity as reflected by LH, FSH, and testosterone concentrations.

Abbreviations: FSH, follicle stimulation hormone; LH, luteinizing hormone; Tes, testosterone.

^a^ *p* < 0.05 compared with other groups.

### 3.8 Oxidative Stress Enzymes

Analysis of oxidative stress markers, including CAT, MDA, GPx, and TAC, revealed the following findings: The highest CAT levels were observed in the CO group, while the lowest were found in the groups receiving high‐ and low‐dose ibuprofen, respectively. This difference was statistically significant (*p* < 0.05). These results suggest that SM extract effectively mitigates oxidative stress, whereas both doses of ibuprofen exacerbate it. Regarding this enzyme, no significant differences were observed between the experimental groups (Table 8). The highest level of MDA was recorded in the IbL group, and the lowest in the SM group; however, this variation was not statistically significant. Comparative analysis of different ibuprofen doses combined with a fixed dose of SM indicated no significant differences in CAT, MDA, or GPx levels when compared to the control group. Ibuprofen appears to induce lipid peroxidation by increasing plasma membrane damage and promoting ROS production, leading to elevated MDA levels. Among all groups, the SM group showed the lowest MDA levels, indicating a protective effect against oxidative damage. The highest GPx activity was observed in the group co‐administered high‐dose ibuprofen and *Salvia miltiorrhiza* extract (SM + IbH), suggesting a compensatory response to counteract ibuprofen‐induced oxidative stress. Overall, administration of SM hydroalcoholic extract demonstrated a significant role in reducing oxidative stress, primarily through enhancement of CAT activity. There were significant differences between the CO group and other groups in TAC enzyme assessment. The highest TAC was in the SM group, and the lowest was in the IbH group (*p < 0.05*).

**TABLE 8:** Malondialdehyde (MDA), glutathione peroxidase (GPX), catalase (CAT), and total antioxidant capacity (TAC) activities in testis tissue.

Groups	Tac (nmol Trolox equivalent/mg‐protein)	Gpx (unit/mg‐pr)	MDA (μmol/mg‐pr)	Cat (unit/mg‐pr)
CO	0.41 ± 0.009	20.54 ± 0.66	0.218 ± 0.022	0.375 ± 0.038
SM	**0.49 ± 0.22** ^a^	20.07 ± 1.59	0.207 ± 0.028	0.364 ± 0.055
IbH	**0.30 ± 0.34** ^a^	21.07 ± 1.01	0.23 ± 0.025	**0.314 ± 0.036** ^a^
IbL	**0.33 ± 0.33** ^a^	19.97 ± 1.19	0.24 ± 0.015	0.342 ± 0.040
SM + IbH	**0.32 ± 0.32** ^a^	21.71 ± 0.75	0.20 ± 0.016	0.345 ± 0.027
SM + IbL	**0.34 ± 0.34** ^a^	21 ± 0.86	0.20 ± 0.016	0.348 ± 0.046

*Note:* These findings suggest that high‐dose ibuprofen may reduce the overall antioxidant capacity in testicular tissue, potentially contributing to oxidative imbalance, although it does not appear to significantly elevate lipid peroxidation.

^a^ *p* < 0.05 compared with CO group.

### 3.9 Histopathological Parameters

The comparative analysis of histopathological parameters showed that the highest mean seminiferous tubular diameter (MSTD) was observed in the control group, while the lowest value was recorded in the SM + IbH group. This difference was statistically significant (*p* < 0.001). Regarding germinal cell thickness (GCCT), the control group exhibited the highest measurement, whereas the lowest value was found in the SM + IbH group (*p* < 0.001). Additionally, the highest number of germinal cell layers (GECE) was observed in the control group, and the lowest in the IbH group. This variation was also statistically significant (*p* < 0.001). Overall, these findings indicate that ibuprofen consumption, particularly at high doses (IbH), leads to significant tissue alterations, including reduced seminiferous tubule diameter and germinal epithelium thickness, as well as a decreased number of germinal cell layers. These changes may ultimately contribute to a reduction in testicular weight (Table 9).

**TABLE 9:** Comparison of pathological parameters.

Groups	MSTD (μm)	GCCT (μm)	GECE (count)
CO	292.85 ± 1.86	78.74 ± 1.85	8.04 ± 0.407
SM	**279 ± 4.59** ^a^	**61.52 ± 1.90** ^a^	**6.11 ± 0.134** ^a^
IbH	**265.79 ± 3.81** ^a^	**60.1 ± 0.75** ^a^	**6 ± 0.057** ^a^
IbL	**267 ± 2.79** ^a^	**60.67 ± 1.15** ^a^	**6.11 ± 0.109** ^a^
SM + IbH	**268.32 ± 3.81** ^a^	**59.91 ± 0.73** ^a^	**6.58 ± 0.508** ^a^
SM + IbL	**273.35 ± 7.67** ^a^	**61.17 ± 1.09** ^a^	**7.01 ± 0.456** ^a^

*Note:* MSTC: The Diameter of Seminiferous, GCCT: Average Thickness, GECE: Number of Cell Layers. These findings indicate that experimental interventions significantly altered the structural organization of seminiferous tubules, potentially affecting spermatogenesis and overall testicular function.

^a^ *p* value < 0.001.

**FIGURE 2:**
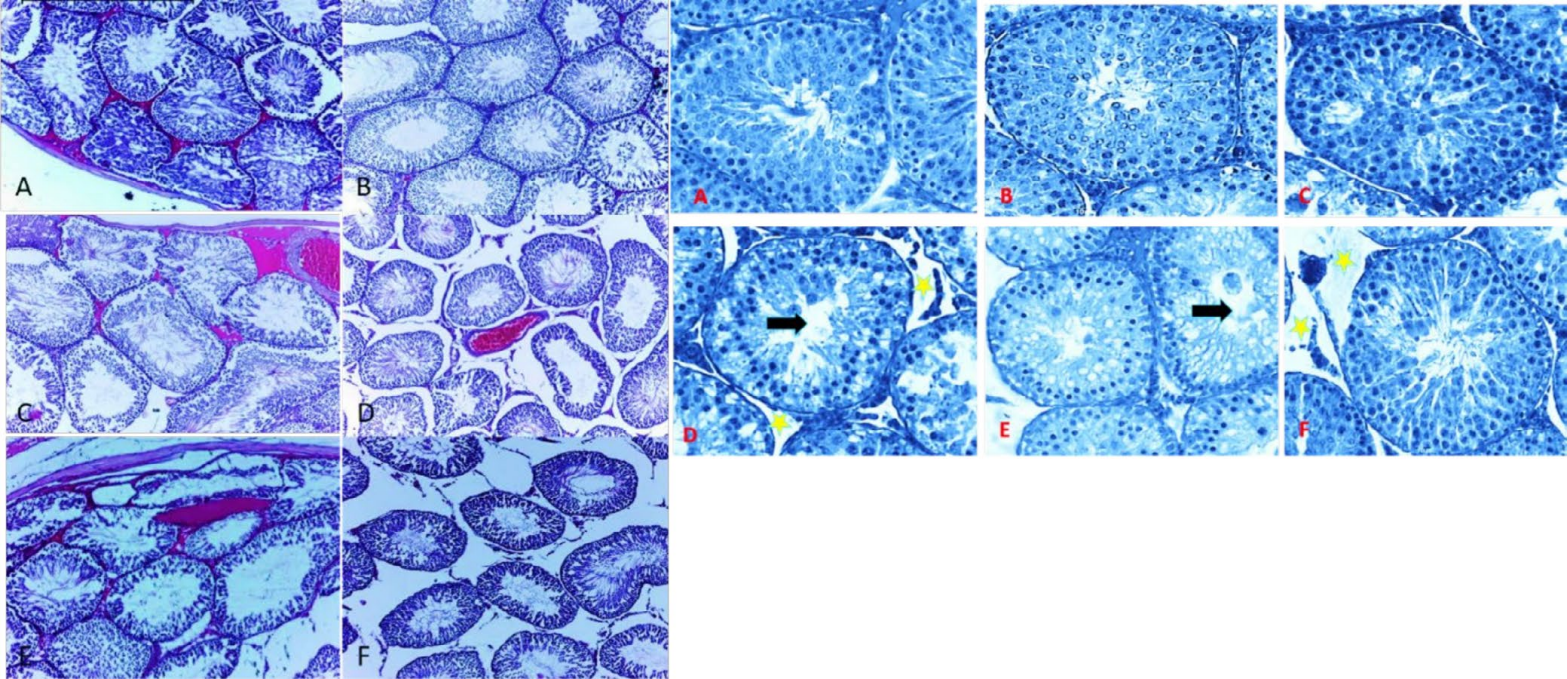
Hematoxylin and eosin (H&E) stained slides of seminiferous tubules in the study groups. (A) Control group (with 400X and 200X magnification): The seminiferous tubules exhibit normal morphology, with spermatogonial cell density within the expected range. (B) SM group: Same the CO group. (C) IBL group: Shows degenerative parts of interstitial space between seminiferous tubules. (D) IBH group: Shows remarkable reduction in the intertubular space and breakdown of the extracellular matrix. (E) SM + IBL group: Shows reduction of mature sperm inside seminiferous tubules (Black arrows). Changing extracellular matrix is not obvious. (F) SM + IBH group: Significant reduction in the intertubular space and degradation of the extracellular matrix (yellow stars' markers).

In the histological evaluation, no significant structural differences were observed between the CO and the SM, indicating that the tissue architecture was preserved in both groups. The seminiferous tubules in cross‐sectional views of these groups exhibited normal morphology, with spermatogonial cell density within the expected range. Additionally, the intertubular spaces remained unchanged without noticeable alterations, suggesting maintenance of tissue homeostasis. In contrast, histopathological changes were observed in the treated groups (IbL and IbH). Particularly in the high‐dose ibuprofen group (IbH), a remarkable reduction in the intertubular space was noted, which may be attributed to degradation or breakdown of the extracellular matrix. Furthermore, in certain regions, signs resembling cellular necrosis were detected, reinforcing the possibility of direct drug‐induced damage to testicular cells. These findings highlight the negative impact of high‐dose ibuprofen on the structural integrity of testicular tissue (Figure 2).

## 4 Discussion

The widespread use of ibuprofen, a non‐steroidal anti‐inflammatory drug (NSAID), has raised concerns regarding its potential adverse effects on male reproductive function. In parallel, the therapeutic potential of natural antioxidants such as *Salvia miltiorrhiza* has been increasingly explored in recent years for their protective role against drug‐induced gonadotoxicity. This study evaluated the impact of hydroalcoholic extract of SM on hormonal and cellular parameters of spermatogenesis in rats following ibuprofen administration. Our findings provide evidence that ibuprofen, particularly at high doses, disrupts testicular function through mechanisms involving oxidative stress and steroidogenic impairment, while SM demonstrates significant protective effects by enhancing antioxidant defenses and preserving spermatogenesis.

Our results demonstrated a significant increase in testicular weight in the SM‐treated group compared to the control group, whereas ibuprofen consumption led to a dose‐dependent reduction. These findings align with previous studies showing that ibuprofen may interfere with signaling pathways involved in germ cell proliferation and Sertoli cell function (Ben Maamar et al. 2017; Hurtado‐Gonzalez et al. 2018; Manku et al. 2020). The observed increase in testicular weight following SM treatment may be attributed to its flavonoid content, including apigenin and quercetin, which are known to promote cell proliferation and reduce oxidative damage (Wu et al. 2019; Brindisi et al. 2021). In contrast, no significant changes were observed in epididymal weight across all groups, suggesting that the primary site of action for both ibuprofen and SM is likely the testis rather than the epididymis. This observation supports some reports indicating that ibuprofen mainly affects early stages of spermatogenesis rather than post‐testicular maturation processes (Bushra and Aslam 2010; Barbosa et al. 2020).

No significant alterations regarding effects of ibuprofen were observed in serum levels of LH or FSH among experimental groups, consistent with findings from Shafigh et al. (2022). However, a notable increase in testosterone levels was observed in the SM‐treated group, reinforcing its role in Leydig cell stimulation and steroidogenesis (Davoodi et al. 2020). This finding corroborates previous research demonstrating that SM enhances testosterone synthesis through upregulation of steroidogenic enzymes and increased cholesterol availability (Salah et al. 2016; Alshubaily and Jambi 2018). Ibuprofen's inhibitory effect on testosterone production has been previously reported and is thought to result from interference with prostaglandin synthesis, endocrine disruption, and direct suppression of Leydig cell function (Capelo et al. 2024; Huang et al. 2025). Our findings confirm this mechanism, as both low‐ and high‐dose ibuprofen groups showed reduced testosterone levels compared to the control and SM groups.

The histopathological findings observed in this study, particularly the structural deterioration of seminiferous tubules following high‐dose ibuprofen administration, are consistent with previous reports indicating the gonadotoxic effects of nonsteroidal anti‐inflammatory drugs (NSAIDs) (Kristensen et al. 2018). Several studies have demonstrated that ibuprofen can induce oxidative stress, testicular toxicity, and germ cell biology in the human fetal testis (Barbosa et al. 2020; Ben Maamar et al. 2017; Hurtado‐Gonzalez et al. 2018), which aligns with our observations. Moreover, the protective effect of SM on testicular architecture in our experimental model is supported by literature showing that its active constituents, such as tanshinone and salvianolic acids, exert potent antioxidant and anti‐inflammatory activities (Luo et al. 2023; Basal et al. 2023). These properties likely contribute to the preservation of germinal epithelium integrity and spermatogenesis, as reported in other studies involving herbal interventions against drug‐induced reproductive toxicity.

Johnson scoring did not show statistically significant differences between the experimental and control groups. This may be attributed to the relatively short duration of the intervention or the compensatory mechanisms of the reproductive system. Similar findings were reported by Shafigh et al., who found no significant changes in Johnson scores following ibuprofen administration at comparable doses.

Sperm motility and viability were significantly improved in the SM‐treated group, whereas ibuprofen, especially at high doses, resulted in higher percentages of immotile and morphologically abnormal sperm. These findings are consistent with previous studies showing that ibuprofen‐induced oxidative stress leads to lipid peroxidation, DNA fragmentation, and impaired mitochondrial function in spermatozoa (Fang and Zhong 2020; Wang et al. 2025). However, some studies present contradictory findings. For certain research has shown that low‐dose ibuprofen does not significantly affect testicular morphology or sperm parameters, suggesting a dose‐dependent effect (Kavoussi et al. 2018). Additionally, one study indicated that ibuprofen may exert protective effects in specific pathological conditions, such as testicular torsion/detorsion models, where it reduces ischemia–reperfusion injury (Dokmeci et al. 2007). These findings suggest that the impact of ibuprofen may vary depending on the experimental model and dosage regimen. SM's beneficial effects on sperm parameters can be attributed to its ability to enhance glutathione peroxidase activity and improve blood flow to testicular tissue, thereby promoting sperm maturation and reducing apoptosis (Brindisi et al. 2021). Additionally, SM's anti‐inflammatory properties may protect the seminiferous epithelium from drug‐induced damage.

This study confirmed that ibuprofen increases oxidative stress, as evidenced by elevated MDA levels and reduced CAT activity. These findings support the hypothesis that ibuprofen exacerbates reactive oxygen species (ROS) production through mitochondrial dysfunction and cyclooxygenase inhibition (Liu 2024; Capelo et al. 2024). Conversely, SM significantly enhanced CAT activity and reduced MDA levels, indicating a robust antioxidant effect. These results are consistent with those of Zhao et al. (Zhao et al. 2019), who demonstrated that SM extract reduces lipid peroxidation and protects against oxidative damage in various tissues. While some studies have reported more pronounced effects of ibuprofen on gonadotropins and sperm quality (Halpern et al. 2020), our findings suggest that the impact may vary depending on dosage, duration of exposure, and specific sensitivity. Similarly, discrepancies in the efficacy of SM across studies may stem from variations in plant extraction methods, dosage regimens, or animal models used.

### 4.1 Limitations

One limitation of the current study is the relatively short duration of treatment, which may not fully capture long‐term effects on fertility. Additionally, the molecular mechanisms underlying SM's protective effects—such as gene expression profiles related to steroidogenesis and oxidative stress—were not explored in depth. Future studies should include longer follow‐up periods and molecular analyses to better understand these pathways.

### 4.2 Recommendations for Future Research

To build upon the findings of this study, future research should:

1. Investigate the effects of SM in combination with other commonly used NSAIDs.
2. Evaluate epigenetic changes in germ cells following exposure to these substances.
3. Assess fertility outcomes and offspring health in treated animals.
4. Explore the potential clinical applications of SM as an adjuvant therapy in patients undergoing long‐term NSAID treatment.

## 5 Conclusion

This study demonstrates that ibuprofen, particularly at high doses, exerts detrimental effects on spermatogenesis through mechanisms involving oxidative stress, disruption of steroidogenesis, and histopathological alterations. In contrast, *Salvia miltiorrhiza* extract exhibits protective properties by enhancing antioxidant defense systems, improving testosterone production, and preserving testicular structure and function. These findings highlight the potential of SM as a natural supplement to mitigate the adverse effects of ibuprofen on male reproductive health.

## Author Contributions

**Omid Rajabzadeh:** conceptualization (equal), methodology (equal), supervision (equal), validation (equal), writing – original draft (equal), writing – review and editing (equal). **Fatemeh Salehi:** data curation (equal), methodology (equal), project administration (equal), writing – original draft (equal), writing – review and editing (equal). **Leila Zarei:** conceptualization (equal), methodology (equal). **Yaser Mokhayeri:** formal analysis (equal), software (equal), validation (equal).

## Conflicts of Interest

The authors declare no conflicts of interest.

## Data Availability

The data that support the findings of this study are available on request from the corresponding author.

## References

[fsn370705-bib-0001] Abu‐Darwish, M. , M. S. Abu‐Darwish , C. Cabral , et al. 2013. “Essential Oil of Common Sage (*Salvia officinalis* L.) From Jordan: Assessment of Safety in Mammalian Cells and its Antifungal and Anti‐Inflammatory Potential.” BioMed Research International 2013: 538940. 10.1155/2013/538940. 24224168 PMC3809930

[fsn370705-bib-0002] Agarwal, A. , N. Parekh , M. K. P. Selvam , et al. 2019. “Male Oxidative Stress Infertility (MOSI): Proposed Terminology and Clinical Practice Guidelines for Management of Idiopathic Male Infertility.” World Journal of Men's Health 37, no. 3: 296–312. 10.5534/wjmh.180064. PMC6704307 31081299

[fsn370705-bib-0003] Ahmadi, R. , S. Balali , P. Tavakoli , M. Mafi , and G. R. Haji . 2013. “The Effect of Hydroalcoholic Leaf Extract of *Salvia officinalis* on Serum Levels of FSH, LH, Testosterone and Testicular Tissue in Rats.” Feyz Medical Sciences Journal 17, no. 3: 225–231.

[fsn370705-bib-0004] Akhigbe, R. E. , T. M. Akhigbe , P. A. Oyedokun , and A. C. Famurewa . 2024. “Molecular Mechanisms Underpinning the Protection Against Antiretroviral Drug‐Induced Sperm‐Endocrine Aberrations and Testicular Toxicity: A Review.” Reproductive Toxicology 128: 108629. 10.1016/j.reprotox.2024.01.004. 38825169

[fsn370705-bib-0005] Alshubaily, F. A. , and E. J. Jambi . 2018. “The Possible Protective Effect of Sage (*Salvia officinalis* L.) Water Extract Against Testes and Heart Tissue Damages of Hypercholesterolemic Rats.” International Journal of Pharmaceutical and Phytopharmacological Research 8, no. 1: 62–68.

[fsn370705-bib-0006] Banihani, S. A. 2019. “Effect of Ibuprofen on Semen Quality.” Andrologia 51, no. 4: e13228. 10.1111/and.13228. 30623461

[fsn370705-bib-0007] Barbosa, M. G. , B. C. Jorge , J. Stein , et al. 2020. “Pre‐Pubertal Exposure to Ibuprofen Impairs Sperm Parameters in Male Adult Rats and Compromises the Next Generation.” Journal of Toxicology and Environmental Health, Part A 83, no. 15–16: 559–572. 10.1080/15287394.2020.1786483. 32615883

[fsn370705-bib-0008] Basal, W. T. , A. M. Issa , O. Abdelalem , and A. R. Omar . 2023. “ *Salvia officinalis* Restores Semen Quality and Testicular Functionality in Cadmium‐Intoxicated Male Rats.” Scientific Reports 13, no. 1: 20808. 10.1038/s41598-023-47629-x. 38012170 PMC10682483

[fsn370705-bib-0009] Ben Maamar, M. , L. Lesné , K. Hennig , et al. 2017. “Ibuprofen Results in Alterations of Human Fetal Testis Development.” Scientific Reports 7: 44184. 10.1038/srep44184. 28281692 PMC5345102

[fsn370705-bib-0010] Brindisi, M. , C. Bouzidi , L. Frattaruolo , et al. 2021. “New Insights Into the Antioxidant and Anti‐Inflammatory Effects of Italian *Salvia officinalis* Leaf and Flower Extracts in Lipopolysaccharide and Tumor‐Mediated Inflammation Models.” Antioxidants 10, no. 2: 311. 10.3390/antiox10020311. 33669555 PMC7922507

[fsn370705-bib-0011] Bushra, R. , and N. Aslam . 2010. “An Overview of Clinical Pharmacology of Ibuprofen.” Oman Medical Journal 25, no. 3: 155. 10.5001/omj.2010.41. 22043330 PMC3191627

[fsn370705-bib-0012] Capelo, M. F. , P. B. Monteiro , and B. M. Anastácio . 2024. “Effects of Major Analgesics on Male Fertility: A Systematic Literature Review.” JBRA Assisted Reproduction 28, no. 2: 331–340. 10.5935/1518-0557.20240020. 38546117 PMC11152418

[fsn370705-bib-0013] Cosentino, M. J. , M. Nishida , R. Rabinowitz , and A. T. Cockett . 1986. “Histopathology of Prepubertal Rat Testes Subjected to Various Durations of Spermatic Cord Torsion.” Journal of Andrology 7, no. 1: 23–31. 10.1002/j.1939-4640.1986.tb01003.x. 3944017

[fsn370705-bib-0014] Davoodi, F. , S. Taheri , A. Raisi , et al. 2020. “Investigating the Sperm Parameters, Oxidative Stress and Histopathological Effects of *Salvia miltiorrhiza* Hydroalcoholic Extract in the Prevention of Testicular Ischemia Reperfusion Damage in Rats.” Theriogenology 144: 98–106. 10.1016/j.theriogenology.2019.11.031. 31927420

[fsn370705-bib-0015] Dokmeci, D. , M. Kanter , M. Inan , et al. 2007. “Protective Effects of Ibuprofen on Testicular Torsion/Detorsion‐Induced Ischemia/Reperfusion Injury in Rats.” Archives of Toxicology 81, no. 9: 655–663. 10.1007/s00204-007-0189-2. 17345063

[fsn370705-bib-0017] Esteves, S. C. 2014. “Clinical Relevance of Routine Semen Analysis and Controversies Surrounding the 2010 World Health Organization Criteria for Semen Examination.” International Brazilian Journal of Urology 40: 433–453. 10.1590/S1677-5538.IBJU.2014.04.03. 25254609

[fsn370705-bib-0018] Fang, Y. , and R. Zhong . 2020. Effects of Oxidative Stress on Spermatozoa and Male Infertility. Free Radical Medicine and Biology. IntechOpen. 10.5772/intechopen.86585.

[fsn370705-bib-0019] Fontana, L. , S. M. Sirchia , C. Pesenti , G. M. Colpi , and M. R. Miozzo . 2024. “Non‐Invasive Biomarkers for Sperm Retrieval in Non‐Obstructive Patients: A Comprehensive Review.” Frontiers in Endocrinology 15: 1349000. 10.3389/fendo.2024.1349000. 38689732 PMC11058837

[fsn370705-bib-0021] Halpern, J. A. , R. J. Fantus , C. Chang , et al. 2020. “Effects of Nonsteroidal Anti‐Inflammatory Drug (Nsaid) Use Upon Male Gonadal Function: A National, Population‐Based Study.” Andrologia 52, no. 4: e13542. 10.1111/and.13542. 32072663

[fsn370705-bib-0022] Huang, W. T. , J. H. Wang , and D. C. Ding . 2025. “Ibuprofen Use and Male Infertility: Insights From a Nationwide Retrospective Cohort Study.” European Journal of Obstetrics & Gynecology and Reproductive Biology 307: 128–133. 10.1016/j.ejogrb.2025.02.001. 39908744

[fsn370705-bib-0023] Hurtado‐Gonzalez, P. , R. A. Anderson , J. Macdonald , et al. 2018. “Effects of Exposure to Acetaminophen and Ibuprofen on Fetal Germ Cell Development in Both Sexes in Rodent and Human Using Multiple Experimental Systems.” Environmental Health Perspectives 126, no. 4: 047006. 10.1289/EHP2307. 29665328 PMC6071829

[fsn370705-bib-0024] Jambor, T. , J. Arvay , E. Ivanisova , et al. 2020. “Investigation of the Properties and Effects of *Salvia officinalis* L. on the Viability, Steroidogenesis and Reactive Oxygen Species (ROS) Production in TM3 Leydig Cells In Vitro.” Physiological Research 69, no. 4: 661–673. 10.33549/physiolres.934457. 32584137 PMC8549893

[fsn370705-bib-0025] Kavoussi, P. K. , M. S. Gilkey , C. Hunn , et al. 2018. “Ibuprofen Does not Have an Adverse Impact on Semen Parameters.” Journal of Assisted Reproduction and Genetics 35, no. 12: 2201–2204. 10.1007/s10815-018-1330-2. 30328572 PMC6289917

[fsn370705-bib-0026] Kristensen, D. M. , C. Desdoits‐Lethimonier , A. L. Mackey , et al. 2018. “Ibuprofen Alters Human Testicular Physiology to Produce a State of Compensated Hypogonadism.” Proceedings of the National Academy of Sciences of the United States of America 115, no. 4: E715–E724. 10.1073/pnas.1715556115. 29311296 PMC5789927

[fsn370705-bib-0027] Liu, Y. 2024. “Recent Progress in Adverse Events of Carboxylic Acid Non‐Steroidal Anti‐Inflammatory Drugs (Cba‐Nsaids) and Their Association With the Metabolism: The Consequences on Mitochondrial Dysfunction and Oxidative Stress, and Prevention With Natural Plant Extracts.” Expert Opinion on Drug Metabolism & Toxicology 20, no. 8: 765–785. 10.1080/17425255.2024.2335843. 38980754

[fsn370705-bib-0028] Lu, Y. , and L. Y. Foo . 2002. “Polyphenolics of Salvia—A Review.” Phytochemistry 59, no. 2: 117–140. 10.1016/S0031-9422(01)00588-0. 11809447

[fsn370705-bib-0029] Luo, L. , J. Xue , Z. Shao , et al. 2023. “Recent Developments in *Salvia miltiorrhiza* Polysaccharides: Isolation, Purification, Structural Characteristics and Biological Activities.” Frontiers in Pharmacology 14: 1139201. 10.3389/fphar.2023.1139201. 36937857 PMC10020221

[fsn370705-bib-0020] Manku, G. , P. Papadopoulos , A. Boisvert , and M. Culty . 2020. “Cyclooxygenase 2 (Cox2) Expression and Prostaglandin Synthesis in Neonatal Rat Testicular Germ Cells: Effects of Acetaminophen and Ibuprofen.” Andrology 8: 691–705. 10.1111/andr.12727. 31705786

[fsn370705-bib-0030] McQuay, H. J. , and R. A. Moore . 2007. “Dose–Response in Direct Comparisons of Different Doses of Aspirin, Ibuprofen and Paracetamol (Acetaminophen) in Analgesic Studies.” British Journal of Clinical Pharmacology 63, no. 3: 271–278. 10.1111/j.1365-2125.2006.02749.x. 16869819 PMC2000740

[fsn370705-bib-0032] Rossitto, M. , C. Marchive , A. Pruvost , et al. 2019. “Intergenerational Effects on Mouse Sperm Quality After In Utero Exposure to Acetaminophen and Ibuprofen.” FASEB Journal 33, no. 1: 339–357. 10.1096/fj.201800792R. 29979629

[fsn370705-bib-0033] Salah, M. , M. Hussein , I. Rana , and L. B. Khalid . 2016. “Effect of *Salvia officinalis* l. (Sage) Aqueous Extract on Liver and Testicular Function of Diabetic Albino Male Rats.” Journal of Babylon University/Pure and Applied Sciences 24, no. 4: 83–90.

[fsn370705-bib-0034] Semet, M. , M. Paci , J. Saïas‐Magnan , et al. 2017. “The Impact of Drugs on Male Fertility: A Review.” Andrology 5, no. 4: 640–663. 10.1111/andr.12335. 28622464

[fsn370705-bib-0035] Shafigh, J. S. , N. Sadeghi , D. Zohrabi , M. Tavalaee , and E. M. H. Nasr . 2022. “The Effect of Ibuprofen on Sperm Parameters, Oxidative Stress and Histology of Mice Testis.” Physiology and Pharmacology 26, no. 1: 111. 10.52547/phypha.26.1.111.

[fsn370705-bib-0036] Sulniute, V. , O. Ragažinskienė , and P. R. Venskutonis . 2016. “Comprehensive Evaluation of Antioxidant Potential of 10 Salvia Species Using High Pressure Methods for the Isolation of Lipophilic and Hydrophilic Plant Fractions.” Plant Foods for Human Nutrition 71: 64–71. 10.1007/s11130-015-0526-42016. 26781308

[fsn370705-bib-0037] Walker, T. 2024. “Progress in the Study of Toxic Effects of Drugs on the Male Reproductive System.” Asia‐Pacific Journal of Pharmacotherapy & Toxicology 4: 76–84.

[fsn370705-bib-0038] Wang, Y. , X. Fu , and H. Li . 2025. “Mechanisms of Oxidative Stress‐Induced Sperm Dysfunction.” Frontiers in Endocrinology 16: 1520835. 10.3389/fendo.2025.1520835. 39974821 PMC11835670

[fsn370705-bib-0039] Wongrakpanich, S. , A. Wongrakpanich , K. Melhado , and J. Rangaswami . 2018. “A Comprehensive Review of Non‐Steroidal Anti‐Inflammatory Drug Use in the Elderly.” Aging and Disease 9, no. 1: 143. 10.14336/AD.2017.0216. 29392089 PMC5772852

[fsn370705-bib-0042] World Health Organization . 2021. “WHO Laboratory Manual for the Examinationand Processing of Human Semen.”

[fsn370705-bib-0040] Wu, B.‐Y. , C. T. Liu , Y. L. Su , S. Y. Chen , Y. H. Chen , and M. Y. Tsai . 2019. “A Review of Complementary Therapies With Medicinal Plants for Chemotherapy‐Induced Peripheral Neuropathy.” Complementary Therapies in Medicine 42: 226–232. 10.1016/j.ctim.2018.12.005. 30670246

[fsn370705-bib-0041] Zhao, W. , Y. Yuan , H. Zhao , Y. Han , and X. Chen . 2019. “Aqueous Extract of *Salvia miltiorrhiza* Bunge‐Radix Puerariae Herb Pair Ameliorates Diabetic Vascular Injury by Inhibiting Oxidative Stress in Streptozotocin‐Induced Diabetic Rats.” Food and Chemical Toxicology 129: 97–107. 10.1016/j.fct.2019.04.033. 31022479

